# Economic Burden of Respiratory Viruses in Latin America and the Caribbean (LAC): A Scoping Literature Review

**DOI:** 10.1111/irv.70148

**Published:** 2025-09-04

**Authors:** Nelson J. Alvis‐Zakzuk, Paula Couto, Jorge H. Jara, Miguel Descalzo, Marc Rondy, Stefano Tempia, Andrea Vicari

**Affiliations:** ^1^ Pan‐American Health Organization Washington DC USA; ^2^ Partnership for International Vaccine Initiative Taskforce for Global Health Atlanta Georgia USA

**Keywords:** economic burden, influenza, LMIC, RSV, SARS‐CoV‐2

## Abstract

**Objective:**

The objective of this study is to summarize the state of knowledge on the economic burden and cost of illness due to influenza, SARS‐CoV‐2, respiratory syncytial virus (RSV), and other respiratory viruses (ORV) in Latin America and the Caribbean (LAC).

**Methods:**

We performed a scoping review across three databases (PubMed‐Medline, Scielo, and Embase) without time restriction, including economic burden and cost‐of‐illness studies. We extracted and analyzed data on publication year, population, study type, perspective, costing techniques, and settings. We reported absolute and relative frequencies to summarize the results. Economic burden estimates were divided by the gross domestic product (GDP) for each country. Costs were converted into 2022 international dollars (PPP).

**Results:**

Overall, 2638 articles were retrieved; we included 44 full texts from 16 LAC countries. Twenty‐four (54.5%) studies focused on influenza, 16 (36.4%) on SARS‐CoV‐2, 3 (6.8%) on RSV, and 1 on ORV. Twenty two (50.0%) focused on cost‐effectiveness (related to vaccination)/cost–benefit analysis, and 17 (38.6%) focused on cost of illness. Most studies (*n* = 33, 75.0%) were conducted from the third‐party perspective. Fifty percent of the studies used a bottom‐up costing technique and 29.6% top‐down. Influenza direct medical costs ranged from I$6.6–I$300.3 for outpatients and I$62.8–I$222,920 for inpatients; for RSV from I$68.3–I$1292; and for SARS‐CoV‐2 between I$69.9 and I$38,039. The total annual costs of the influenza economic burden ranged between 0.0003% and 1.33% of the GDP.

**Conclusion:**

This study showed variability in costing methods, perspectives, and types of studies among LAC countries. This variability underscores the need for standardized methodologies in future cost studies to ensure comparability and reliability of results.

## 1 Introduction

Pandemics pose major health and economic challenges worldwide [1]. Influenza and COVID‐19 pandemics have challenged the response capacity of governments, health systems, infrastructures, supply chains, surveillance systems, vaccination programs, and human resources [2]. The Pandemic Influenza Preparedness (PIP) [3] framework was adopted in 2011 to improve global readiness for influenza pandemics by ensuring fair access to vaccines and other pandemic‐related resources [3]. This preparedness and response framework enables the Pan American Health Organization (PAHO) to increase its support for the regional severe acute respiratory infection network (SARI*net*). Countries are supported in enhancing their laboratory, surveillance, and burden of disease (BoD) estimation capacities under the World Health Organization (WHO) Global Influenza Surveillance and Response System (GISRS). In addition, Member States in the Americas are supported by the use of standardized tools for influenza and other respiratory viruses (ORV) analyses and to improve the understanding of the effectiveness of vaccination interventions and their potential economic impac.

Seasonal influenza and ORV epidemics are responsible for considerable disease burden [4]. Each year in the Americas, there are > 700,000 influenza‐associated respiratory hospitalizations [5]. Respiratory syncytial virus (RSV) is responsible for 3.6 million hospital admissions in children aged < 5 years worldwide [6, 7, 8, 9, 10]. Between January 2020 and January 2024, more than 6.8 million COVID‐19‐related deaths have been registered worldwide; from the 20 countries with the highest COVID‐19‐related mortality rates per 100,000 inhabitants, four are from Latin America and the Caribbean (LAC): (Perú—the highest globally—[4.9]; México [4.5]; Brazil [1.9]; and Chile [1.2]) [11]. In addition to the BoD, the economic burden associated with respiratory viruses further impacts health systems, families, and societies globally [12, 13, 14]. For instance, in the United States, the average annual total economic burden of seasonal influenza had a cost for the health system and society of US$11.2 billion (0.35% of United States per capita health expenditure) in 2015 [15]. A study conducted on RSV burden among 72 current and former GAVI‐eligible countries estimated 20.8 million cases, 1.8 million hospitalizations, and 1.2 million disability‐adjusted life years (DALYs), with a total average direct cost of US$ 611 million for 2022 (US$ 509 per DALY) [16].

A 2019 survey indicated a lack of data on the cost‐effectiveness of influenza vaccination programs as a key barrier to initiating and expanding influenza vaccination programs in low‐ and middle‐income countries [17]. Cost‐effectiveness analyses (CEA) and other economic evaluations can provide important information to guide evidence‐based decisions, resource allocation, and long‐term investment in vaccination and other new product introduction approaches by demonstrating value for money. Whereas studies on cost‐of‐illness, economic burden, and economic evaluations of health interventions for treating and preventing disease due to respiratory viruses have been conducted in LAC countries, the types of studies and methods used are poorly understood. Better understanding the landscape of economic burden studies in LAC is a priority for PAHO to further support informed decision‐making on the prevention and control of respiratory viruses. We aimed to conduct a scoping review of the existing literature to provide an overview of studies and methods used to estimate the economic burden or perform economic evaluations (partial or complete) associated with respiratory viruses (influenza, SARS‐CoV‐2, RSV, and ORV) and assess remaining gaps in LAC countries.

## 2 Methods

### 2.1 Literature Search

For this scoping review, we conducted an online search in PubMed‐Medline, Embase, and SciELO according to the PRISMA guidelines [18] with no time restriction. We used search strings with Boolean operators to retrieve the most relevant articles (Table S1).

### 2.2 Eligibility Criteria

We included studies meeting the following eligibility criteria: economic burden studies, partial (cost of illness, cost descriptions, or cost analysis), and complete economic evaluations (CEA mainly for vaccines or cost–benefit analysis [CBA]) [19], published in Spanish, English, Portuguese, or French, and conducted in LAC countries. We included studies on influenza, SARS‐CoV‐2, RSV, or ORVs infection; severe acute respiratory infection (SARI); acute respiratory infection (ARI); or influenza‐like illness cases that reported cost of illness (direct medical, nonmedical, or indirect costs) or an estimation of the economic burden for a municipality, state, region, or country due to the viruses aforementioned. Single‐country studies or those performed in multiple countries that included at least one LAC country were considered. Additionally, we reviewed references from the included studies to further screen relevant literature for inclusion. We excluded articles that did not present original or peer‐reviewed results (i.e., [systematic] literature reviews, conference abstracts, editorials, and commentaries). We did not contact the study authors to request additional data.

### 2.3 Data Extraction and Analysis

Two reviewers independently reviewed the literature and screened titles and abstracts; a third reviewer resolved discrepancies. Information on publication year, type of virus (influenza, SARS‐CoV‐2, RSV, ORVs), study type (partial or complete economic evaluations), type of cost (direct medical, direct nonmedical, and indirect cost), age groups (children, adults, and older adults), study setting—hospital—(multicenter and single center), perspective (third payer, patient/family, and society), level of care (inpatient and outpatient), costing technique method (bottom‐up, top‐down, and others; Tables S2, S8), cost source, national economic burden (if reported), and cost currency (year of costing) were considered for the analysis. CEA and CBA were reviewed to extract respiratory virus cost of illness or economic burden estimates.

For costs extraction, we preferentially abstracted mean values; if not available, we gathered median costs. For country‐specific reports, monetary units were converted to purchasing power parity (PPP) and adjusted to 2022 international dollars (I$) using the following formula:

(1) $$\text{Cost}\;I{\text{\$}}_{\mathrm{PP}P_{2022}}=\text{Cost}\_{\text{country}}_{{\mathrm{LCU}}_{\text{reported year}}}x\left(\frac{\mathrm{CP}I_{{\mathrm{LCU}}_{2022}}}{\mathrm{CP}I_{{\mathrm{LCU}}_{\text{reported year}}}}x\frac{1}{\mathrm{PP}P_{2022}}\right)$$

where *LUC* is the local currency units, *CPI* is the Consumer Price index, and *PPP* is the purchasing power parity conversion factor.

We first identified the cost and currency defined in each study, and then, we converted the reported costs into value in local currency within the studies' registered exchange rate. Next, we adjusted these costs to 2022 local currency using the historical consumer price index (CPI) of each country and converted them to 2022 PPP adjusted I$ using conversion rates from the International Monetary Fund registered in the World Bank (WB) databases [20]. Specific CPI data were obtained from national statistical bureaus or central banks when countries' data were not available in the registered CPI WB databases, such as Argentina [21] and Cuba [22]. Likewise, for Cuba, we assumed the 2011 PPP conversion factor was constant through 2022 because it was the only available data for PPP equivalence in this country. Additionally, we utilized the WB data to obtain the gross domestic product (GDP) of LAC countries and estimated the gross national economic burden, when reported, as a percentage of each country's GDP. Economic burden was reported in 2022 PPP using the aforementioned conversion.

A descriptive analysis was performed. Absolute and relative frequencies were used to describe the study features. Ranges of costs were used to report direct medical, nonmedical direct, and indirect costs by age groups and type of virus. We used Rayyan as software to develop the selection of the papers and evidence to be included and Zotero as the reference manager of the study. We gathered and analyzed the data from the included articles using a standardized form in Microsoft Excel and R programming using dplyr, ggplot2, and tidyplots packages.

## 3 Results

**FIGURE 1:**
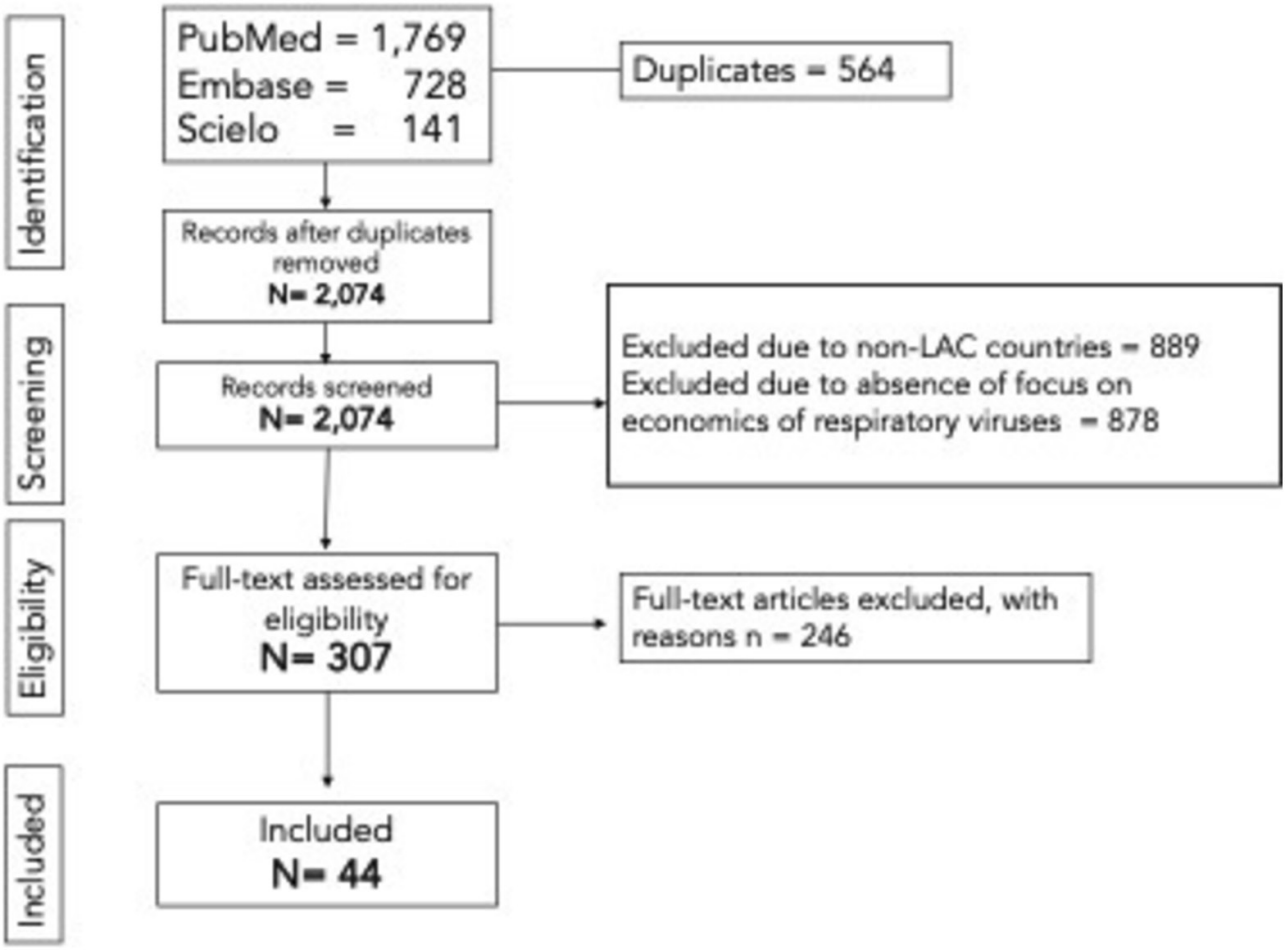
Flow diagram of the scoping review results of economic cost studies of influenza, COVID‐19, RSV, and other respiratory viruses in Latin America and the Caribbean.

Our search yielded 2,638 publications. After removing duplicates (564) and screening for titles and abstracts (2074), 307 articles were reviewed in their full‐text versions. We included 44 full‐text studies that met our inclusion criteria (Figure 1). More than half of the articles were published in 2020–2023 (*n* = 24; 54.5% of the total) (Figure S1).

Most studies included were on influenza (*n* = 23: 52.3%) and SARS‐CoV‐2 (*n* = 16: 36.4%), conducted in a single country (*n* = 42: 95.5%), and most of them focused on cost‐effectiveness/cost–benefit analysis (*n* = 23: 52.3%). The studies typically used the third payer perspective (*n* = 33; 75.0%) were conducted in single settings (*n* = 19: 43.2%), and 23 (52.3%) studies focused only on inpatients (Tables 1 and S3). The costing approach used a bottom‐up methodology in 22 (50.0%) studies and a top‐down approach in 20 (45.5%). For influenza and SARS‐CoV‐2, other methodologies, such as surveys and the value of statistical life, were used to estimate cost of illness (Tables 1 and S3).

**TABLE 1:** Characteristics of economic cost studies of influenza, COVID‐19, RSV, and other respiratory viruses in Latin America and the Caribbean (*n* = 44).

Main characteristics	Description	*N*	%
Type of virus	Influenza	23	52.3%
	SARS‐CoV‐2	16	36.4%
	RSV	3	6.8%
	Influenza, RSV, & ORV	1	2.3%
	ORV	1	2.3%
Type of country study	Multicountry	2	4.5%
	Single country	42	95.5%
Study type	Cost‐effectiveness	22	50.0%
	Cost of illness	17	38.6%
	Economic burden of disease	3	6.8%
	Budget impact analysis	1	2.3%
	Cost–benefit	1	2.3%
Study perspective	Third payer	27	61.4%
	Society	6	13.6%
	Society and third payer	6	13.6%
	Patient/family	3	6.8%
	Hospital	2	4.5%
Method	Bottom‐up	15	34.1%
	Top‐down	13	29.5%
	Bottom‐up/top‐down	7	15.9%
	Standard costing	5	11.4%
	Survey	3	6.8%
	Value of statistical life	1	2.3%
Setting	Single setting	19	43.2%
	Multicenter	11	25.0%
	NA	14	31.8%
Level of care	Inpatient	23	52.3%
	Both	20	45.5%
	Outpatient	1	2.3%

We found eligible studies published from 16 out of 33 LAC countries. The included studies were conducted in Argentina, Bolivia, Brazil, Colombia, Costa Rica, Chile, Cuba, Ecuador, El Salvador, Haiti, Honduras, Mexico, Nicaragua, Panama, Peru, and Uruguay. The countries with the most publications were Colombia (12), Brazil (11), and Argentina (10). Economic evaluations for RSV and ORV (including rhinovirus, human metapneumovirus, parainfluenza viruses 1–3, and adenovirus) were carried out only in Argentina, El Salvador, Nicaragua, Panama, and GAVI countries (including Haiti). The highest number of studies by country for influenza and RSV was from Argentina, and for SARS‐CoV‐2, it was from Colombia and Brazil (Figure S2).

Forty‐two studies (95.5%) reported direct medical costs, representing 47.8% of influenza studies, 81.3% of SARS‐CoV‐2, and 100% of RSV and ORV studies (Table S4 and Figure S3). Indirect costs were reported in a smaller fraction of studies (16 out of 44 or 36.4%), with 4.3% related to influenza and 6.3% related to SARS‐CoV‐2. Out‐of‐pocket (OOP) health expenditures were described in five studies (11.4%), mainly for influenza (17.4%, *n* = 4), and one OOP estimation was performed for influenza, RSV, and ORV [23] (Table S4 and Figure S3).

Costs of illness for outpatient and inpatient episodes of influenza, SARS‐CoV‐2, RSV, and ORV are provided in Table 2 and Figures S4–S6. Direct medical costs for inpatients showed great variability in the analyzed studies by countries and age groups (Figures S4 and S5). Mexico reported the highest cost for hospitalization episodes for influenza (I$222,919) and Brazil for SARS‐CoV‐2 (I$ 38,039) (Table 2 and Figure S4). Influenza outpatient direct medical costs ranged from I$7 to I$300 [24, 27, 28, 29, 30, 31, 32, 33, 34, 35, 36, 37, 38]; RSV was from I$69 to I$28,616; and SARS‐CoV‐2 did not present costs solely based on outpatient costs. Indirect costs and OOP health expenditures were reported for all studied viruses. For influenza, indirect costs due to productivity losses varied from I$12 to I$1719 (Figure S6); indirect costs due to mortality were estimated only in Argentina, reporting a loss of I$27,340 owing to premature deaths [36]; OOP ranged from I$3 to I$97 [24, 25]. For SARS‐CoV‐2, indirect costs (productivity losses due to morbidity and mortality) ranged from I$1773 to I$22,908 [47, 56, 59], and OOP was calculated in I$96 [47]. For ORV, indirect costs varied from I$27 to I$250 [23]. Costs were mainly reported in children (*n* = 25: 56.8%) for all analyzed viruses (Table 2).

**TABLE 2:** Minimum and maximum costs by viruses, type of costs and age groups* reported in the economic cost studies of influenza, COVID‐19, RSV, and other respiratory viruses in Latin America and the Caribbean (values expressed in I$).

Type of viruses	Type of cost	Outpatient	Children^a^	Adults^a^	Older adults^a^	All	Total	References
		Inpatient						
Influenza	OOP	Inp/out	—	17	—	7	(7–17)	[24, 25]
		Inpatient	97	—	—	63	(63–97)	[25, 26]
		Outpatient	—	—	—	(3–12)	(3–12)	[25]
	Direct medical	Inpatient	(100–222,920)	(112–7160)	(675–28,392)	(704–20,070)	(100–222,920)^b^	[26, 27, 28, 29, 30, 31, 32, 33, 34, 35, 36, 37, 38, 39, 40, 41, 42, 43, 44]
		Outpatient	(18–300)	(42–335)	269	(7–119)	(7–300.3)	[24, 27, 28, 29, 30, 31, 32, 33, 34, 35, 36, 37, 38]
	Productivity loss	Inp/out	23	—	—	112	(23–112)	[29, 31, 35, 36, 45, 46]
		Inpatient	(13–140)	—	—	—	(13–140)	
		Outpatient	(12–37)	(258–1719)	—	—	(12–1719)	
	Indirect due to disability	Inp/out	—	1711	—	40	(40–1711)	[24, 25, 26, 35, 36]
		Inpatient	174	—	—	(254–728)	(174–728)	
		Outpatient	—	—	—	(31–364)	(31–364)	
	OOP & indirect	Inpatient	—	—	—	(413–416)	(413–416)	[25, 40]
		Outpatient	—	—	—	70	70	
	Indirect due to mortality	Inp/out	—	—	—	27,340	27,340	[36]
	Economic	Inp/out	(355–955)	—	—	—	(355–955)	[23, 26, 40]
		Inpatient	3864	—	—	(2395–3864)	(2395–3864)	
SARS‐CoV‐2	Direct medical	Inp/out	—	(4383–8606)	—	—	(4383–8606)	[47, 48, 49, 50, 51, 52, 53, 54, 55]
		Inpatient	(70–20,870)	(248–38,039)	(1835–34,759)	(947–33,381)	(70–38,039)	
		Selfcare	—	—	—	100	100	
	Direct non‐medical	Inp/out	—	96	—	—	96	[47]
	Productivity loss	Inp/out	—	(1773–4128)	—	—	(1773–4128)	[47, 56]
	Indirect cost due to mortality	Inpatient	—	—	—	(19,312–22,908)	(19,312–22,908)	[57]
RSV	Direct medical	Inpatient	(195–1292)	—	—	—	(195–1292)	[16]
		Outpatient	(68–286)	—	—	—	(68–286)	
	Economic	Inp/out	(752–1123)	—	—	—	(752–1123)	[23]
ORV	OOP	Inp/out	(32–98)	—	—	—	(32–98)	[23]
		Inpatient	(26–119)	—	—	—	(26–119)	
		Outpatient	(19–22)	—	—	—	(19–22)	
	Productivity loss	Inpatient	(27–33)	—	—	—	(27–33)	
	OOP & indirect	Inpatient	(148–250)	—	—	—	(148–250)	
	Direct medical	Inpatient	(567–5603)	—	—	—	(567–5603)	[23, 58]
	Economic	Inp/out	(568–1772)	—	—	—	(568–1772)	[23]
		Inpatient	(710–5988)	—	—	—	(710–5988)	

Abbreviation: Inp/out, inpatients and outpatients.

^a^ Older adults: > 60 years old; children: < 18 years old; adults: 18–59 years old; all: no restriction of age, could include children, adults, and older adults; total: summarize all age categories.

^b^ Results from a Mexican study on inpatients with comorbidities.

Seventeen of the 44 studies reported the economic burden of influenza, SARS‐CoV‐2, or ORV in the general population by age group (Table 3). Total annual costs of the economic burden of influenza, as a proportion of the GDP, ranged from 0.0003% (Peru [25], OOP in adults) to 1.33% (Argentina [31], economic costs in children inpatients) of the national GDP. For indirect costs of influenza, the economic burden varied from 0.008% (Peru [25], in children) to 0.55% (Argentina [31], in children). The economic burden estimated for SARS‐CoV‐2 ranged from 0.002% (OOP in Colombian [47] adults) to 4.78% (direct costs in Colombian [48] adults). Burden related to indirect costs ranged from 0.0009% in Equatorian [56] health workers to 17.95% in Peru [25], in the general population. For ORV, the economic burden expressed in direct cost was 0.0194% in the Nicaraguan [58] inpatient children.

**TABLE 3:** Economic burden associated to the analyzed viruses by age group, hospital setting, type of cost and perspective in the economic cost studies of influenza, COVID‐19, RSV, and other respiratory viruses in Latin America and the Caribbean (values expressed in I$).

Virus	Country	Hospital setting	Perspective	Study period	Type of cost	Age group	Value	% of GDP
Influenza	Colombia [34]	Inpatient	Third payer	2007	DC	< 2 years	$566,617	0.0002%
					DC		$2,575,903	0.0007%
					DC	> 65 years without vaccine	$142,691,171	0.0415%
					DC	All	$343,133,199	0.0999%
	Colombia [26]	Inpatient	Society	2014	DC	< 5 years	$102,315,004	0.0298%
					DC		$20,028,736	0.0058%
					EC		$122,343,741	0.0356%
	Peru [25]	Both	Patient/family	2009–2010	DC	< 5 years	$9,418,020	0.0039%
						5–17 years	$10,866,946	0.0045%
					IC	< 5 years	$15,696,700	0.0065%
						5–17 years	$93,455,740	0.0385%
					EC	< 5 years	$31,393,401	0.0129%
						5–17 years	$108,186,489	0.0446%
					DC	18–49 years	$5,312,729	0.0022%
						50–64 years	$724,46	0.0003%
					IC	18–49 years	$39,603,982	0.0163%
						50–64 years	$7,003,143	0.0029%
					EC	18–49 years	$50,229,441	0.0207%
						50–64 years	$8,693,557	0.0036%
					DC	> 65 years	$724,46	0.0003%
					IC	> 65 years	$4,105,290	0.0017%
					EC	> 65 years	$7,003,143	0.0029%
	Peru [25]	Inpatient	Patient/family	2009–2010	EC	All	$205,747,520	0.0848%

	Brazil [29]	Outpatient	Society	2010–2017	DC	All	$14,144,150	0.0007%
		Inpatient			DC		$30,008,476	0.0016%
		Outpatient			IC		$206,118,750	0.0107%
		Inpatient			EC		$358,204,413	0.0187%
	Brazil [30]	Inpatient	Third payer	2015–2020	DC	All	$12,412,644,740	0.6465%
	Argentina [31]	Both	Society	1998	DC	< 17 years	$4,884,250,649	0.7739%
					IC		$3,488,750,417	0.5528%
					EC		$8,373,001,066	13,267%
	Argentina [32]	Both	Third payer	2020	DC	> 65 years	$435,864,192	0.0691%
		Inpatient					$3,155,336	0.0005%
		Both					$74,139,139	0.0117%
	Argentina [35]	Inpatient	Society and third payer	2021	DC	All	$76,602,080	0.0121%
		Inpatient			DC		$143,300,224	0.0227%
	Chile [39]	Inpatient	Third payer	2009–2010	DC	15+ years	$33,693,336	0.0112%
Mexico [43]	Inpatient	Third payer	2004	DC	> 65 years	$7,104,350,000	0.4847%
SARS‐COV‐2	Brazil [48]	Inpatient	Third payer	2021	DC	18+ years	$35,099,716,531	1.8280%
	Argentina [48]						$7,381,570,435	1.1696%
	Colombia [48]						$16,439,852,667	4.7843%
	Costa Rica [48]						$2,017,268,458	2.9133%
	Peru [48]						$7,822,174,666	3.2239%
	Chile [48]						$3,057,276,640	1.0156%
	Mexico [48]						$52,103,880,063	3.5545%
	Ecuador^a^ [56]	Both	Patient/family	2020	IC	20+ years	$1,019,470	0.0009%

	Colombia^a^ [47]	Both	Society	2020	DC	18+ years	$378,570,137	0.1102%
		Both			DC	18+ years	$8,244,466	0.0024%
		Both			IC	18+ years	$661,977,184	0.1926%
		Both			EC	18+ years	$1,046,793,982	0.3046%
	Mexico [49]	Inpatient	Third payer	2020–2021	DC	All	$1,109,227,677	0.0757%
	Peru [57]	Inpatient	Society	2020–2021	IC	All	$43,529,149,061	17.9404%
		Inpatient					$17,556,756,816	7.2360%
		Inpatient					$25,972,392,246	10.7045%
	Argentina [60]	Inpatient	Third payer	2021–2022	DC	All	$6,968,148,767	1.1041%
	Mexico [60]	Inpatient			DC	All	$6,687,102,457	0.4562%
	Brazil [60]	Inpatient			DC	All	$16,820,242,582	0.8760%
	Colombia [60]	Inpatient			DC	All	$7,110,133,337	2.0692%
	Peru [60]	Inpatient			DC	All	$10,641,966,867	4.3861%
Chile [60]	Inpatient	DC			All	$12,239,285	0.0041%
Other viruses	Nicaragua [58]	Inpatient	Third payer	2009–2011	DC	6 months to 9 years	$3,046,959	0.0194%

Abbreviations: DC, direct cost; EC, economic cost; IC, indirect cost.

^a^ Estimation in health workers.

## 4 Discussion

To the best of our knowledge, this is the first review that describes cost of illness and economic burden studies for various respiratory viruses in LAC, showing high variability in the state of knowledge. Out of the 33 LAC countries (660.6 million inhabitants), 16 countries, accounting for 89.0% of the population, were represented in the 44 studies reviewed. Although this proportion of countries with studies could be considered substantial, a review of the literature from high‐income countries reveals that this proportion approaches nearly 100% [61, 62]. These findings underscore the necessity for additional research encompassing diverse populations, particularly in middle‐ and low‐income countries (LMIC). In this scoping review, most of this evidence was published during pandemic periods, in 2012 and between 2020 and 2023, likely driven by the need for surveillance and cost evidence following the 2009 influenza A(H1N1)pmd09 pandemic and the 2020 SARS‐CoV‐2 pandemic [63].

Our results show that influenza virus cost‐of‐illness studies span across all types of estimates, with estimates on direct medical and nonmedical costs and indirect costs. By contrast, we did not find studies that provided information on OOP health expenditures and indirect costs for RSV in LAC. Other systematic reviews found a lack of available estimates of healthcare resource utilization in LMIC settings; most studies were conducted in the United States [64]. Another systematic review conducted in 2013 of cost of illness and CEA for influenza analyzed 140 studies globally [62], of which only eight were from LAC. In our review, we found 24 studies of this nature, showing an increase in the economic evidence for influenza. A recent systematic review published in 2024 on influenza illness [61] found 10 cost‐of‐illness and CEA studies in LMIC from 2012 to 2022. For the same period, and using a similar search strategy, our review found 12.

Previous similar literature reviews focused mainly on influenza [62, 65]; we added SARS‐CoV‐2, RSV, and ORV. As these studies found, published studies analyzed mostly direct medical costs. OOP health expenditures and indirect costs, both generally assumed by the patients or their families, were less studied [62, 65]. Estimates of direct medical costs from the health system perspective are important to efficiently allocate healthcare budgets. However, understanding the cost incurred by the patients and families provides a broader perspective of the impact of respiratory viruses and improves their economic burden estimates. For example, a recent literature review on influenza in LMICs concluded that influenza and acute respiratory infections impose a substantial economic burden (catastrophic costs), particularly from hospitalizations, on the lowest income households [66].

Cost‐of‐illness analyses and economic burden estimates can be performed using several methodologies and costing techniques. Understanding the importance of measuring the economic impact of respiratory viruses in LMIC, in 2015, the WHO released the Manual for Estimating the Economic Burden of Seasonal Influenza [67] as technical guidance to support countries with a methodological framework. However, only five of the 25 influenza studies included in this review referenced this manual. We also found a high variability in costing methodologies throughout the revised studies; for instance, half of the studies used more precise methods, such as bottom‐up costing, whereas others used less accurate methods, making it difficult to compare and synthesize the study's results. This finding highlights the need for standardization of costing methods [65, 68] and displays a gap between the formulation of technical recommendations and their applications in LMIC, such as most in LAC. The release of updated, user‐friendly tools is essential for standardizing the estimations in LAC countries, alongside comprehensive training to facilitate their application.

Our results showed the proportional impact on the economic productivity of several LAC countries. Most countries with available estimates have an economic impact on their productivity of < 1% of the GDP. By contrast, economic burden estimates > 1% were described for Argentina, Brazil, Colombia, and Peru. Specifically, the economic burden of influenza and SARS‐CoV‐2 could reach up to 17% of the GDP of Peru. Efforts to mitigate the costs of influenza, SARS‐CoV‐2, and ORV can potentially alleviate this impact. Providing national policymakers with economic burden estimates allows for the informed prioritization of health resources and enhances epidemic and pandemic preparedness and responses.

Our study has several limitations. Our research focused on peer‐reviewed journals accessible through PubMed‐Medline, Scielo, and Embase. We may have missed nonpeer‐reviewed studies published in local or national journals. The absence of publications in 17/33 LAC countries does not mean that studies have not been performed; further review of the gray literature using unconventional data sources could enrich this scoping review. Second, our search strategy did not consider nonrespiratory outcomes; methods for estimating the economic burden of nonrespiratory outcomes due to respiratory viruses are still in progress. Finally, we found high variability in costing methods, data inputs, and costing perspectives in the included studies. To ensure better comparability and allow for data pooling, cost collection and evaluation tools at different levels, including local, national, regional, and among population groups, clustering categories, and various components of direct and indirect costs are key inputs to consider when developing local manuals [65].

Our review highlights the crucial role of political commitment and organized national immunization strategies in the implementation of influenza vaccination initiatives across Latin America. Countries that have incorporated influenza vaccination into their frameworks—often with guidance from technical advisory groups and support from the PAHO Revolving Fund—demonstrate enhanced surveillance, better data reporting, and increased research output [69]. Political will, evidenced by the prioritization of specific demographics and the establishment of regular campaigns (e.g., Vaccination Week in the Americas), enhances pandemic preparedness and leads to more economic burden studies [69]. Conversely, nations lacking robust policy mandates or stable immunization systems face obstacles in sustaining vaccination efforts and generating local evidence. This disparity underscores the link between political and structural factors and the quality of economic evaluations, thereby impacting regional equity in vaccine access and decision‐making.

Further cost‐of‐illness studies and economic burden estimations should be conducted in LAC countries, particularly for RSV and ORV. Partial economic evaluations such as cost‐of‐illness analysis are mandatory inputs to conduct complete economic evaluations (CEA/CBA) for vaccines and health technologies of interventions that might mitigate the disease burden of respiratory viruses. In addition to guidelines, tools, and manuals that standardize cost‐of‐illness estimation in low and middle‐income settings, strengthening existing health technology assessment (HTA) agencies and launching new ones will complement and promote the implementation of studies related to economic evaluations in LAC countries. New HTA agencies in countries where no economic studies have been conducted are a necessity to increase economic burden estimates for respiratory viruses in LAC. In conclusion, there have been important progresses in LAC to estimate the economic burden of influenza, SARS‐CoV‐2, RSV, and ORV. However, standard approaches applied more systematically in a broader range of countries are key to improving cost‐evidence‐based decisions in countries lacking evidence. Enhancing regional networks and knowledge transfer, along with the adoption of best practices, can provide better support for these efforts.

## Author Contributions

**Nelson J. Alvis‐Zakzuk:** investigation, writing – original draft, writing – review and editing, data curation, formal analysis, visualization, methodology, conceptualization, validation. **Paula Couto:** writing – original draft, writing – review and editing, validation, methodology, supervision, project administration, investigation, conceptualization. **Jorge H. Jara:** conceptualization, writing – review and editing, writing – original draft, formal analysis, methodology, validation, investigation. **Miguel Descalzo:** writing – original draft, writing – review and editing, visualization, methodology, investigation. **Marc Rondy:** writing – review and editing, visualization, project administration, supervision, validation, writing – original draft. **Stefano Tempia:** writing – review and editing, validation, funding acquisition, writing – original draft. **Andrea Vicari:** writing – review and editing, project administration, supervision, investigation, funding acquisition.

## Conflicts of Interest

The authors declare no conflicts of interest.

## Peer Review

The peer review history for this article is available at https://www.webofscience.com/api/gateway/wos/peer‐review/10.1111/irv.70148.

## Supporting information


**Table S1:** Electronic search strategy used in this literature scoping review.
**Table S2:** Costing techniques methods.
**Table S3:** Methodology approaches implemented in the included studies by type of virus.
**Table S4:** Studies included in the systematic review of the literature and main findings.
**Figure S1:** Absolute and cumulative frequencies of manuscripts on the economic burden of COVID‐19, influenza, RSV, and ORV in Latin America and the Caribbean per year of issue.
**Figure S2:** Number of manuscripts by countries and type of virus included in this review.
**Figure S3:** Proportion of studies reporting direct, out‐of‐pocket, and indirect costs by type of virus.
**Figure S4:** Direct medical costs* reported by LAC countries and respiratory viruses. Costs expressed in PPP, 2022.
**Figure S5:** Violin plots of direct medical costs reported by LAC countries, age groups, and respiratory viruses. Costs expressed in PPP, 2022.
**Figure S6:** Indirect costs reported by LAC countries and respiratory viruses. Costs expressed in PPP, 2022.

## Data Availability

The data that support the findings of this study are available on request from the corresponding author.
