# Supplementary material for: Economic Burden of Respiratory Viruses in Latin America and the Caribbean (LAC): A Scoping Literature Review
Source: Influenza Other Respir Viruses. 2025 Sep 4;19(9):e70148. doi: 10.1111/irv.70148 (PMC12411011; doi:10.1111/irv.70148)
Supplement: Supplementary file 1 — Table S1: Electronic search strategy used in this literature scoping review. Table S2: Costing techniques methods. Table S3: Methodology approaches implemented in the included studies by type of virus. Table S4: Studies included in the systematic review of the literature and main findings. Figure S1: Absolute and cumulative frequencies of manuscripts on the economic burden of COVID‐19, influenza, RSV, and ORV in Latin America and the Caribbean per year of issue. Figure S2: Number of manuscripts by countries and type of virus included in this review. Figure S3: Proportion of studies reporting direct, out‐of‐pocket, and indirect costs by type of virus. Figure S4: Direct medical costs* reported by LAC countries and respiratory viruses. Costs expressed in PPP, 2022. Figure S5: Violin plots of direct medical costs reported by LAC countries, age groups, and respiratory viruses. Costs expressed in PPP, 2022. Figure S6: Indirect costs reported by LAC countries and respiratory viruses. Costs expressed in PPP, 2022. [file IRV-19-e70148-s001.docx]

**Supplementary material**

**Table 1S. Electronic search strategy used in this literature scoping review.**

| **Date** | **Database** | **Search strategy** |
| --- | --- | --- |
| **11/01/2023** | **PubMed – Medline** | ("influenza"[Title] OR "covid-19"[Title] OR "sars-cov-2"[Title] OR "respiratory syncytial virus"[Title] OR "respiratory virus"[All Fields]) AND ("economic burden"[Title] OR "cost"[Title] OR "cost of illness"[MeSH Terms]) |
| **11/01/2023** | **Embase** | (influenza:ti OR 'coronavirus disease 2019':ti OR 'severe acute respiratory syndrome coronavirus 2':ti OR 'respiratory syncytial virus':ti OR 'respiratory virus':ti) AND ('economic burden':ti OR cost:ti OR 'cost of illness':ab,ti) |
| **11/01/2023** | **Scielo** | ((cost) AND ((covid-19) OR (influenza) OR (syncytial respiratory virus))​ |

**Table 2S. Costing techniques methods.**

| Bottom-Up | Costs by aggregating individual components or activities. It starts from the smallest unit of analysis (e.g., specific treatments or procedures) and sums up the costs to arrive at the total cost.  ﻿It is a micro-costing approach where resource data are collected for each individual patient. Typically, a sample of patients from a population is selected, and a set of resources is identified to be recorded during their care pathway for a determined period. The measurement of the resources will be in natural units: time of health workers, units of medicines, time use of room, items of medical equipment, and so on^1^. |
| --- | --- |
| Top-down | This approach estimates costs by using aggregate data and applying cost estimates to overall categories or departments. It often involves allocating indirect costs based on broad categories or averages. ﻿  Estimate mean costs for the full set of products and services (cost objects) produced by the organization during a given period.  In top-down, data are collected at the organizational level, e.g., cost center.  Could be general (Gross-costing) or detailed (Micro-costing), in ﻿Activity‑based costing (ABC) studies or traditional top-down detailed studies^1^ |
| Bottom-up/Top-down | Hybrid method combines elements of both bottom-up and top-down approaches. It uses detailed data from the bottom-up approach for specific components and incorporates broader cost estimates from the top-down approach for overall cost allocation^1^. |
| Standard costing | Standard costing involves assigning a predetermined cost to each unit of service or product based on historical data, clinical experts' practice or experience, guidelines, or institutional standards of practice. These standard costs are then compared against actual costs to identify variances. |
| Survey | This method involves collecting cost data through surveys or questionnaires distributed to healthcare providers, patients, or other stakeholders. It gathers information on costs from those directly involved or affected. |
| Value of statistical life (VSL) | VSL, as the human capital method calculates, is based on the cost that generates a premature death for society, that is the loss of an individual's productive potential (material loss) through the present value of their future income.  It is a method used to estimate the monetary value of reducing the risk of death or improving health outcomes. It is based on the amount people are willing to pay for improvements in health. |

**Table 3S. Methodology approaches implemented in the included studies by type of virus.**

| Method | Influenza (n=22) | SARS-CoV-2 (n=16) | RSV  (n=3) | Influenza, RSV & ORV (n=2) | ORV  (n=1) |
| --- | --- | --- | --- | --- | --- |
| Bottom-up | 11 (45.8%) | 8 (44.4%) | - | 3 (100%) | 22 (44.9%) |
| Top-down | 4 (16.7%) | 4 (22.2%) | 2 (50.0%) | - | 10 (20.4%) |
| Standard costing | 2 (8.3%) | 1 (5.6%) | 2 (50.0%) | - | 5 (10.2%) |
| Survey | 3 (12.5%) | 1 (5.6%) | - | - | 4 (8.2%) |
| Bottom-up/top-down | 3 (12.5%) | 1 (5.6%) | - | - | 4 (8.2%) |
| N/A | 1 (4.2%) | 1 (5.6%) | - | - | 2 (4.1%) |
| Value of statistical life | - | 2 (11.1%) | - | - | 2 (4.1%) |

**Table 4S. Studies included in the systematic review of the literature and main findings.**

| Author (year) | Country | Virus | Perspective | Type of costs | Principal results |
| --- | --- | --- | --- | --- | --- |
| Dayan (2001) ^2^ | Argentina | Influenza | Society | Direct and indirect costs | Vaccination would lead to net savings of US$ 11894870 per vaccinated cohort (US$ 10.04 per vaccinated child). |
| Fariña (2002) ^3^ | Argentina | SRV | Third payer | Direct costs | Hospitalization cost = $184 777. Palivizumab administration cost would have been $185 064. |
| Morales (2004) ^4^ | Colombia | Influenza | Patient/family | Indirect costs | The employer can save US$6.4 to US$25.8 per vaccinated employee based on labor costs alone. |
| Gutiérrez (2005) ^5^ | Mexico | Influenza | Third payer | Direct costs | Net cost per life year saved between 13 301 and 21 037 Mexican pesos (about $1 210 and $1 910 US dollars). |
| Sousa (2005) ^6^ | Brazil | SARS-CoV-2 | Hospital | Direct costs | Average total cost per hospitalization = US$11 260 (R$63 504) for COVID-19 patients; in only detectable cases, the value was US$17178 (R$96886). |
| Rodríguez (2008) ^7^ | Argentina | SRV | Third payer | Direct costs | For high-risk patients, one averted hospitalization was associated with costs of U.S. dollars (US$)13 198 number needed to treat (NNT): 4.5 |
| Porras-Ramírez (2009) ^8^ | Colombia | Influenza | Third payer | Direct costs | Flu vaccination ICER among children <2 ranged from US$ 1,900 to US$ 2,967 per averted death. ICER was cost-saving in adults aged over 65. |
| Mota (2011) ^9^ | Brazil | Influenza | Hospital | Direct and indirect costs | Costs during May– October reached R$798,051.87 (≈US $443,362). |
| Giglio (2012) ^10^ | Argentina | Influenza | Third payer | Direct costs | Flu total incremental cost of hospital admissions and outpatient consultations = $ 91512 (2009). |
| Armstrong (2012) ^11^ | Chile | Influenza | NR | Direct costs | Intensive/intermediate hospital charges = US$ 20,304 and US$ 1,262 (general wards). We estimated US$ 20 million in hospital charges for flu-related hospitalizations during the first wave for the whole country. |
| Basurto-Dávila R (2012) ^12^ | Argentina | Influenza | Patient/family | Direct and indirect costs | School closures significantly impact low-income households. Authorities should consider the range of economic impacts of school closures on families when planning their implementation. |
| Chocontá-Piraquive (2012) ^13^ | Colombia | Influenza | Third payer | Direct costs | The costs of care avoided would be US$249 530 to US$842 163. For the baseline scenario, vaccinating pregnant women would be cost-effective (ICER/APVP avoided US$7 657). |
| Alvis-Guzmán (2015) ^14^ | Nicaragua | ORV | Third payer | Direct costs | Total average cost of healthcare cases = US$314.9 and US$971.60 for people in ICU. |
| Gibson (2016) ^15^ | Brazil | Influenza | NR | Direct costs | Incremental costs per QALY for Brazil were £2,817. |
| Tinoco (2016) ^16^ | Peru | Influenza | Society | Direct, out-of-pocket, and indirect costs | Confirmed influenza illness paid a median of $13 for self-treatment, $19 for ambulatory non-medical-attendance illness, $29 for ambulatory medical-attendance illness, and $171 for hospitalizations. |
| Lara (2018) ^17^ | Colombia | Influenza | Society and third-payer | Direct and indirect costs | ICER = $1,280 per (DALY) averted, the total incremental cost of the vaccination program would be $776,800, and it would avert four deaths and 332 DALYs for the five cohorts. |
| Van Bellinghen (2018) ^18^ | Brazil | Influenza | Society | Direct, out-of-pocket, and indirect costs | The discounted cost-utility ratio of quadrivalent versus trivalent influenza vaccine was R$20,428 with FLOU, R$22,768 with FLORA), and R$19,257 with FLORENCE. |
| Vecoso (2019) ^19^ | Brazil | Influenza | Third payer | Direct costs | Cost for chemoprophylaxis was BRL 39.42; ambulatory care, BRL 12.47; hospital admission, BRL 5,727.59); ICU, BRL 19,217.25 (SD 7,917.33); and adverse events, BRL 292.05 ICER = BRL –4,080.63 (US$ –1,263.74)/QALY and –982.39 (US$ –304.24)/H1N1 prevented. |
| Salcedo-Mejía (2019) ^20^ | Colombia | Influenza | Society | Direct, out-of-pocket, and indirect costs | Median DMC hospitalized case in general ward = $743.50 and in ICU $4669.80. The economic cost per hospitalized case was $1826.10; DMC = 93.8% of this cost. The median indirect cost was $82.10 and the median OutOP per case was $45.70. |
| Jara (2019) ^21^ | El Salvador | ORV | Society and third-payer | Direct, out-of-pocket, and indirect costs | In El Salvador, direct and indirect costs per illness episode =US$38 (IQR 22–72); median government-paid hospitalization cost = US$118 (IQR 59–384); overall societal cost of US$219 (IQR 101–416) per severe ARI episode. |
| Jara (2019) ^21^ | Panama | ORV | Society and third payer | Direct, out-of-pocket, and indirect costs | In Panama, direct and indirect costs = US$75 (IQR 39–135), and the healthcare system paid US$280 (IQR 150–420) per hospitalization, producing an overall societal cost of US$393 (IQR 258–552) |
| Tenorio-Mucha (2020) ^22^ | Peru | SARS-CoV-2 | Third payer | Direct costs | On average, drug prices in private pharmacies are 11 times higher than in public pharmacies. |
| Sánchez Infante (2020) ^23^ | Cuba | SARS-CoV-2 | Third payer | Direct costs | ARI medical care in the emergency service costs 104 441,16 CUP (Cuban Pesos), and acute otitis media costs 24 036,22 CUP. |
| Fernandes (2020) ^24^ | Brazil | SARS-CoV-2 | Third payer | Direct costs | The vaccines showed incremental cost-utility ratios ranging from R$223161.3/QALY (Oxford) to R$17757.85/QALY (CoronaVac). |
| Crépey (2020) ^25^ | Brazil | Influenza | Society | Direct, out-of-pocket, and indirect costs | 3400 life-years saved for an incremental direct cost of R$169 million per year, down to R$86 million from a societal perspective. ICER for the switch would be R$49,700 per life-year saved and R$26,800 per QALY gained from a public payer perspective. |
| Falcón-Lezama (2020) ^26^ | Mexico | Influenza | Society | Direct and indirect costs | Overall, a decrease in the economic burden for the Mexican HCS of US $111.9 million |
| Li, Xiao (2020) ^27^ | GAVI countries | SRV | Third payer | Direct costs | The optimal strategy of choice tends to change for increasing WTP values per DALY averted from the current situation to maternal vaccination (at WTP > US$1000) to mAB (at WTP > US$3500). |
| Miethke-Morales (2021) ^28^ | Brazil | SARS-CoV-2 | Third payer | Direct costs | The average cost of the 3254 admissions (51.7% ICU) was US$12,637.42. |
| ﻿Sánchez (2021) ^29^ | Cuba | SARS-CoV-2 | Third payer | Direct costs | The average cost per salary was higher in nursing (226,2 CUP), and medications were generated at a high cost (35,6 %). |
| Rojas (2021) ^30^ | Mexico | SARS-CoV-2 | Third payer | Direct costs | The measures' benefits are 526 billion US$. Under a plausible economic recovery trajectory, the net cost of mitigation in terms of the output gap was equal to 35% of Mexico’s 2019 GDP. |
| Morillo (2021) ^31^ | Ecuador | SARS-CoV-2 | Patient/family | Direct and indirect costs | As a result, 145 workers were isolated for COVID-19 for a total of 2312 days, thus generating a direct cost for the Institution of $98,097.14 for absence from work. |
| Urueña (2021) ^32^ | Argentina | Influenza | Society and third payer | Direct and indirect costs | ICER per QALY was 13,590 and 11,678 US$ from the payer’s and societal perspectives, respectively. |
| Baral (2021) ^33^ | GAVI countries | RSV | Third payer | Direct costs | ICER per DALY averted = US$1342 (range, US$800–US$1866) for maternal RSV vaccine and US$431 (range, US$167–US$692) for mAbs. |
| ﻿Castillo-Rodríguez (2022) ^34^ | Colombia | Influenza | Society and third payer | Direct, out-of-pocket, and indirect costs | Media direct medical cost per influenza patient = US$ 700. Median OOPE and indirect costs per patient were US$ 147 (95% CIUS$94–202. Total costs were US$ 848 (95% CI US $ 646–1,011). |
| ﻿Curro-Urbano (2022) ^35^ | Peru | SARS-CoV-2 | Society | Indirect costs | From the start of the pandemic until July 31, 2021, the social cost of deaths due to COVID-19 has amounted to US$ 18,807,942,464.6. |
| ﻿Alvis-Zakzuk (2022) | Colombia | SARS-CoV-2 | Third payer | Direct costs | The median cost for the ICU was $4118 (IQR 2069-5455), three times higher than for those hospitalized only in the general ward. |
| ﻿Reyes-Lopez (2022) ^36^ | México | Influenza | Third payer | Direct costs | Subjects admitted at the PICU generated the highest cost (mean $29,608.62 US$), followed by patients over age 10 (mean $49,674.53 US$). |
| ﻿Taborda (2022) ^37^ | Multicounty | SARS-CoV-2 | Third payer | Direct costs | The different COVID-19 vaccination schemes generate potential savings ranging from US$100 million to US$1.5 billion per country. |
| Morales-Zamora (2022) ^38^ | Colombia | SARS-CoV-2 | Third payer | Direct costs | An increment of US$255 million results in an ICER of US$3339 per DALY avoided. The no-prioritization strategy is still cost-effective, with an ICER of US$5223.66. |
| Urueña (2022) ^39^ | Argentina | Influenza | Society and third payer | Direct and indirect costs | From the payers' perspective, the ICER per QALY gained was US$12,214 in the base case and US$ 2311 in the high egg-adaptation scenario. |
| Bianculli (2022) ^40^ | Uruguay | Influenza | Society and third payer | Direct and indirect costs | The incremental cost per QALY gained with QIV would be US$18,000 for both the payor and societal perspectives, for all age groups, and around US$12,000 for adults ≥65 years of age. |
| Guzmán (2022) ^41^ | Colombia | SARS-CoV-2 | Society and third payer | Direct and indirect costs | From the social and health systems perspectives, the program saves an average of $1,045 and $850 per case, respectively. |
| ﻿Prada (2023) ^42^ | Colombia | SARS-CoV-2 | Third payer | Direct costs | The costs in the HFOT group were US$ $7992, and in the COT group, US$ $10,190 (9402). |
| ﻿Reyes-Lopez (2023) ^43^ | México | SARS-CoV-2 | Third payer | Direct costs | The estimated average cost per patient was US$5,943 (95% CI: US$4,249–7,637). When the costs of the three clinical areas were summed, only the 5–to 10-year-old group showed a maximum cost of US$14,000. |
| ﻿Torres-Toledano (2023) ^44^ | México | SARS-CoV-2 | Third payer | Direct costs | The average cost of care per patient was US$ 6,557 ± 4,997. |
| Augustovski (2023) ^45^ | Multicountry | SARS-CoV-2 | Third payer | Direct costs | The realistic (base case) vaccination campaign in Chile was the only scenario. Although it was not cost saving, it was still highly cost‐effective, with an ICER of US$22 per QALY gained. |
| Oliveira (2023) ^46^ | Brazil | SARS-CoV-2 | Third payer | Direct costs | The median cost per admission was estimated at US$359.78 and US$1385.80 for the SUS and hospital perspectives, respectively. In addition, patients who stayed between 1 and 4 days in (ICU had 60.9% higher costs than non-ICU patients. |
| Wang (2023) ^47^ | Colombia | SARS-CoV-2 | Society | Direct and indirect costs | Total economic losses attributable to SARS-CoV-2 infection among HCWs as a share of total health expenditure ranged from 1.51% in Colombia to 8.38% in Western Cape province, South Africa |

**Figure 1S. Absolute and cumulative frequencies of manuscripts on the economic burden of COVID-19, influenza, RSV, and ORV in Latin America and the Caribbean per year of issue.**

**Figure 2S. Number of manuscripts by countries and type of virus included in this review.**

**Figure 3S. Proportion of studies reporting direct, out-of-pocket, and indirect costs by type of virus.**

**Figure 4S. Direct medical costs* reported by LAC countries and respiratory viruses. Costs expressed in PPP, 2022**


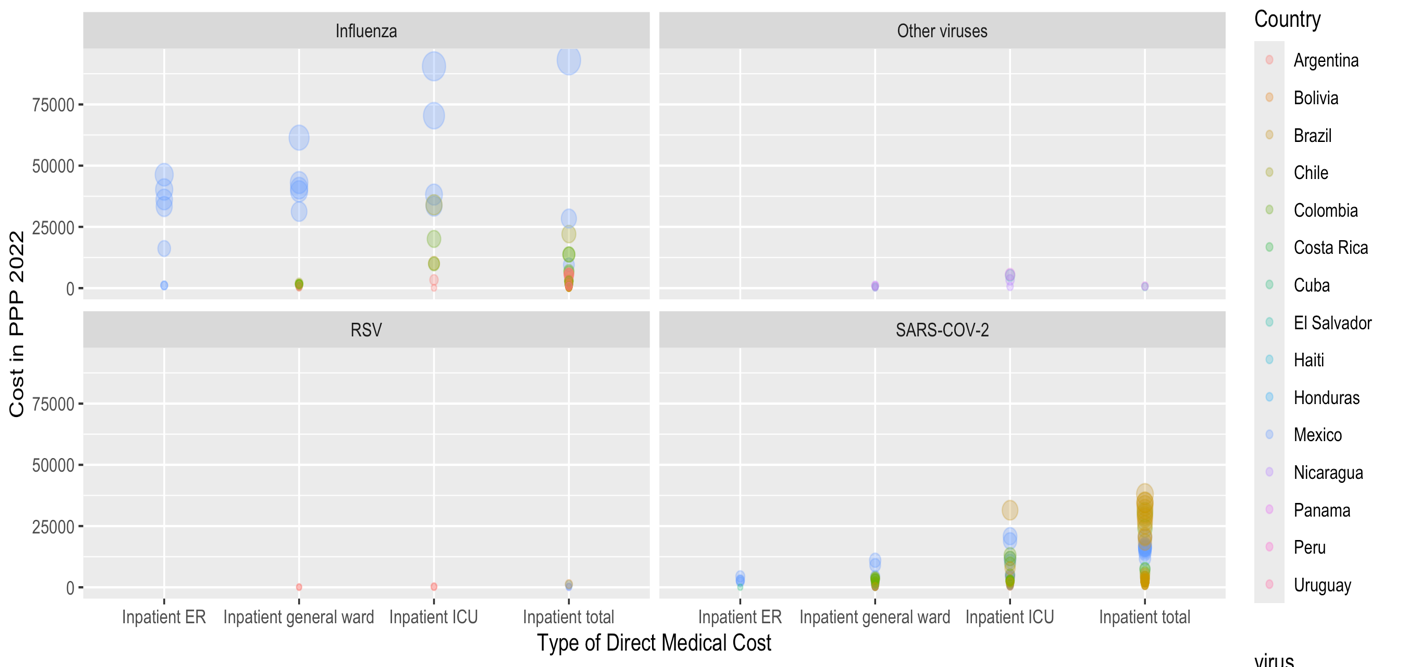


*Extreme data wasn’t included, censured to cost<I$100,000

**Figure 5S. Violin plots of direct medical costs reported by LAC countries, age groups, and respiratory viruses. Costs expressed in PPP, 2022**

**
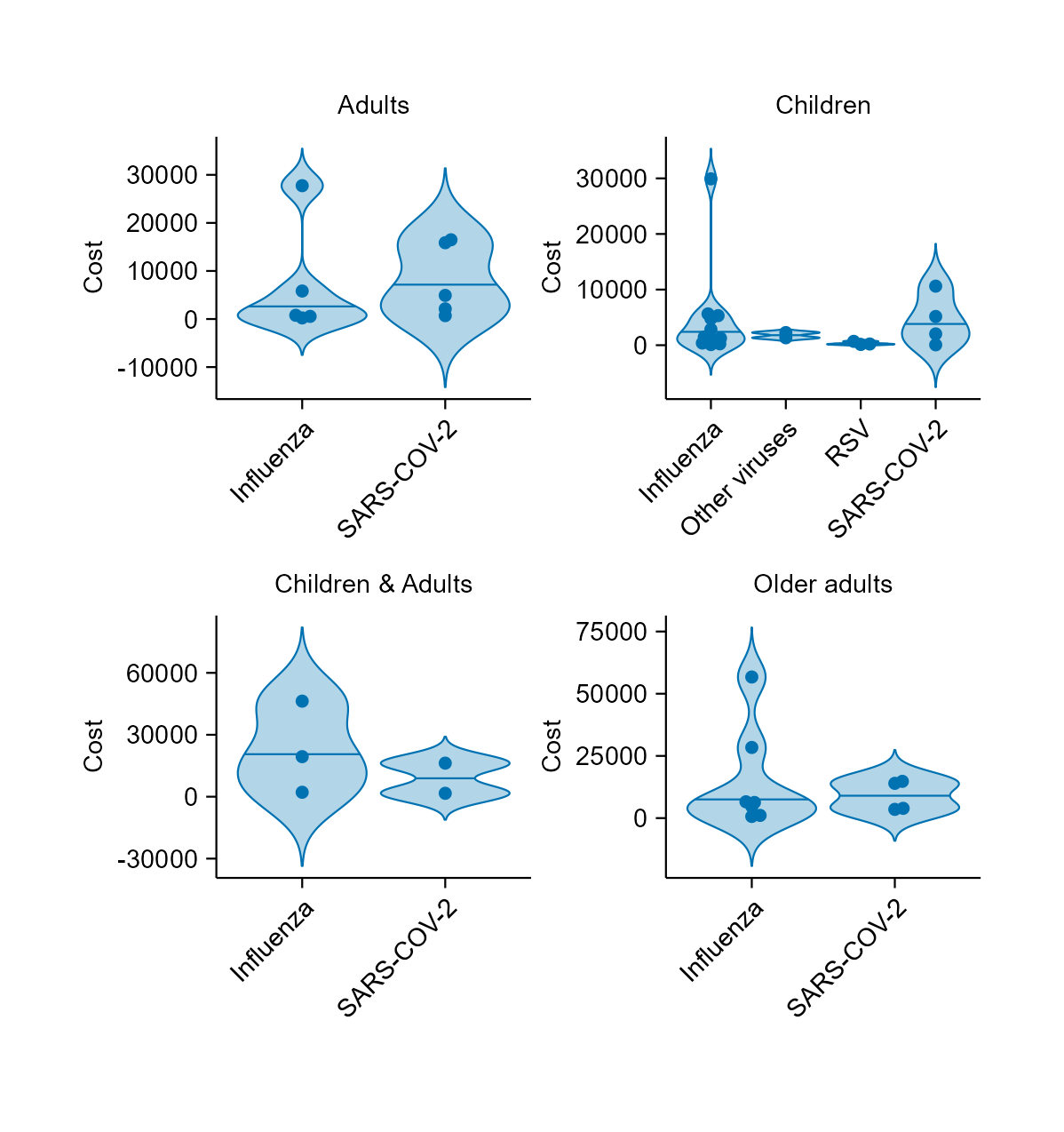
**

**Figure 6S. Indirect costs reported by LAC countries and respiratory viruses. Costs expressed in PPP, 2022**

**
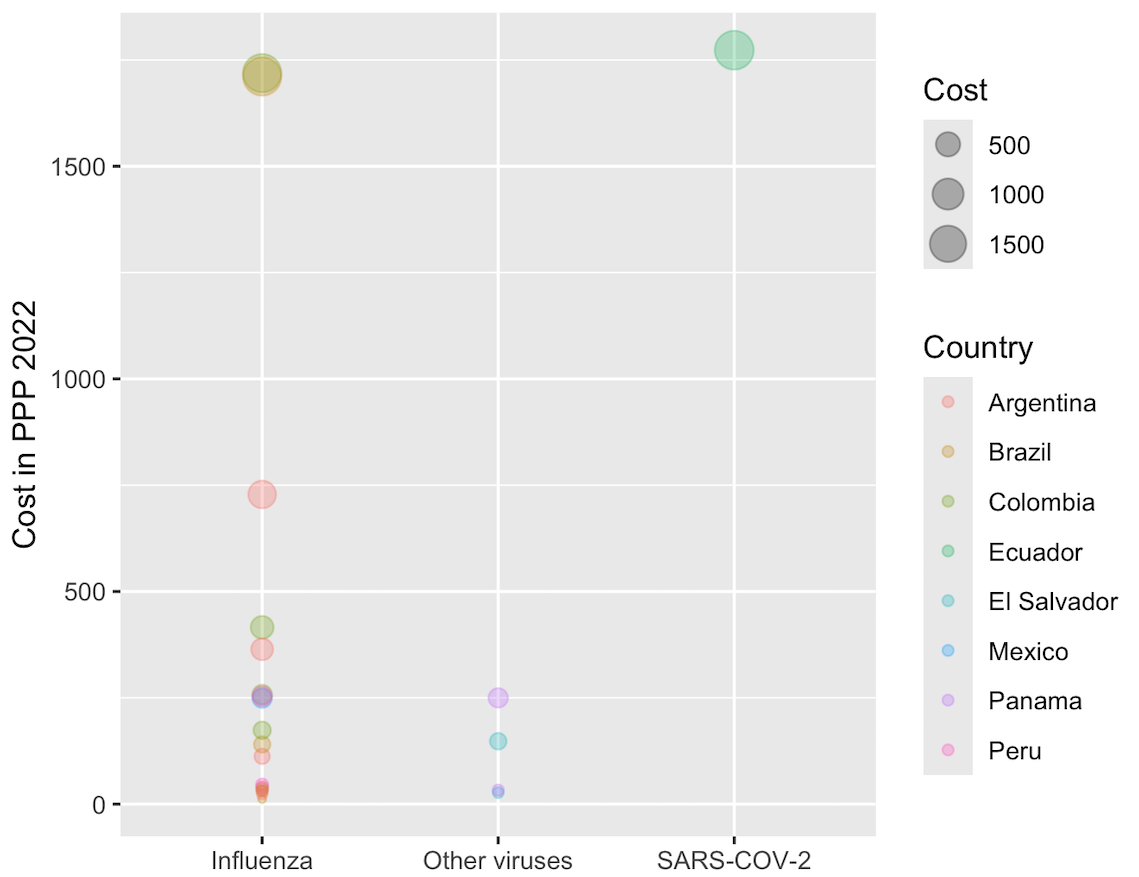
**

**References**

1. Špacírová Z, Epstein D, García-Mochón L, Rovira J, Olry de Labry Lima A, Espín J. A general framework for classifying costing methods for economic evaluation of health care. *Eur J Health Econ*. 2020;21(4):529-542. doi:10.1007/s10198-019-01157-9

3. Fariña D, Rodríguez SP, Bauer G, et al. Respiratory syncytial virus prophylaxis: cost-effective analysis in Argentina. *Pediatr Infect Dis J*. 2002;21(4):287-291. doi:10.1097/00006454-200204000-00006

7. Rodríguez SP, Fariña D, Bauer G. Respiratory syncytial virus prophylaxis in a high-risk population in Argentina: a cost-effectiveness analysis. *Pediatr Infect Dis J*. 2008;27(7):660-661. doi:10.1097/INF.0b013e3181691753

12. Basurto-Dávila R, Garza R, Meltzer MI, et al. Household economic impact and attitudes toward school closures in two cities in Argentina during the 2009 influenza A (H1N1) pandemic. *Influenza Other Respir Viruses*. 2013;7(6):1308-1315. doi:10.1111/irv.12054

22. Tenorio-Mucha J, Lazo-Porras M, Hidalgo AM, Málaga G, Cárdenas MK. Precios de medicamentos esenciales para el manejo y tratamiento de la COVID-19 en establecimientos farmacéuticos peruanos públicos y privados. *Acta Médica Peruana*. 2020;37(3):267-277. doi:10.35663/amp.2020.373.1560

24. Fernandes RRA, Santos M da S, Magliano CA da S, et al. Cost Utility of Vaccination Against COVID-19 in Brazil. *Value Health Reg Issues*. 2022;31:18-24. doi:10.1016/j.vhri.2022.01.009

28. Miethke-Morais A, Cassenote A, Piva H, et al. COVID-19-related hospital cost-outcome analysis: The impact of clinical and demographic factors. *Braz J Infect Dis*. 2021;25(4):101609. doi:10.1016/j.bjid.2021.101609

30. Rojas Valdés RI. The economic benefits and costs of COVID-19 mitigation measures in Mexico. *Estudios Económicos (México, DF)*. 2021;36(1):27-56. doi:10.24201/ee.v36i1.415

33. Baral R, Higgins D, Regan K, Pecenka C. Impact and cost-effectiveness of potential interventions against infant respiratory syncytial virus (RSV) in 131 low-income and middle-income countries using a static cohort model. *BMJ Open*. 2021;11(4):e046563. doi:10.1136/bmjopen-2020-046563

42. Prada SI, Garcia-Garcia MP, Ospina-Tascón GA, Rosselli D. Cost Analysis of High-Flow Oxygen Therapy Compared with Conventional Oxygen Therapy in Severe COVID-19 in Colombia: Data from a Randomized Clinical Trial. *Clinicoecon Outcomes Res*. 2023;15:733-738. doi:10.2147/CEOR.S412087
